# Supplementary material for: Mesenchymal Stem Cells and Begacestat Mitigate Amyloid-β 25–35-Induced Cognitive Decline in Rat Dams and Hippocampal Deteriorations in Offspring
Source: Biology (Basel). 2023 Jun 25;12(7):905. doi: 10.3390/biology12070905 (PMC10376406; doi:10.3390/biology12070905)
Supplement: Supplementary file 1 [file biology-12-00905-s001.zip › biology-2422715-supplementary.pdf]

PND7
Replicate 1
APP
p-Tau

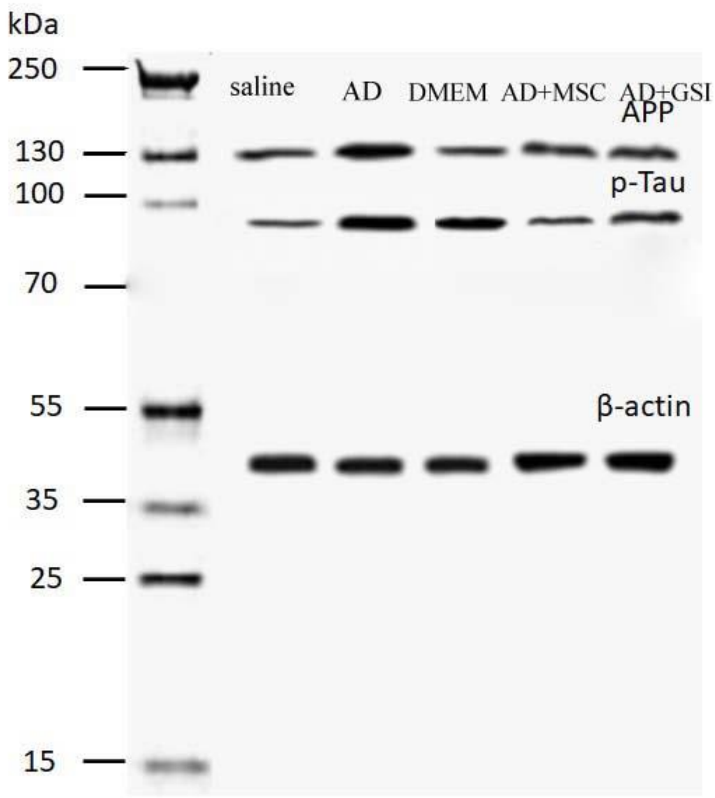

Figure S1: Original immunoblot (replicate 1) of APP and p-Tau of hippocampus at PND7

PND14
Replicate 1
APP
p-Tau

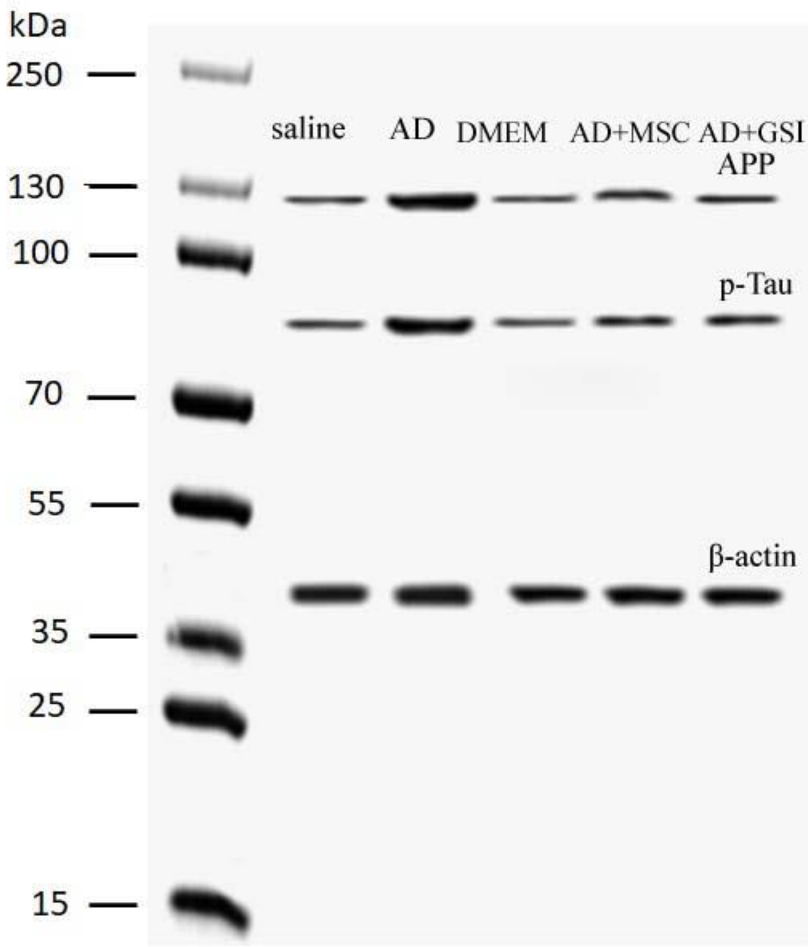

Figure S2: Original immunoblot (replicate 1) of APP and p-Tau of hippocampus at PND14

PND21
Replicate 1
APP
p-Tau

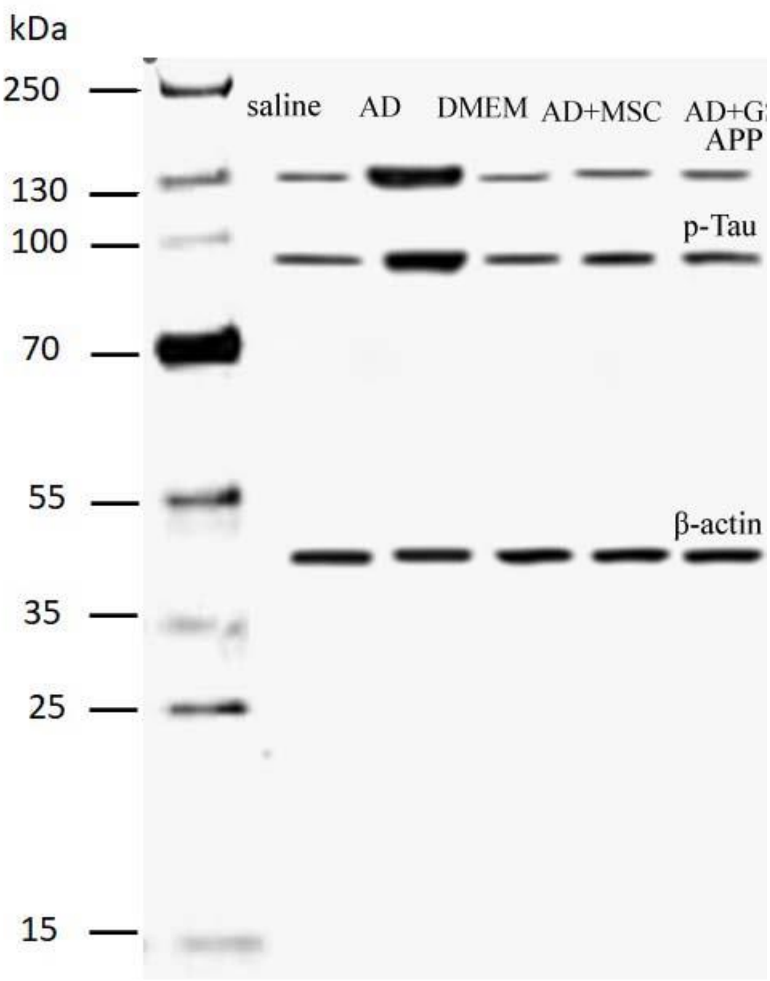

Figure S3: Original immunoblot (replicate 1) of APP and p-Tau of hippocampus at PND21
